# Molecular mechanisms underlying increased radiosensitivity in human papillomavirus-associated oropharyngeal squamous cell carcinoma

**DOI:** 10.7150/ijbs.40880

**Published:** 2020-02-04

**Authors:** Huanhuan Wang, Bin Wang, Jinlong Wei, Lingbin Meng, Qihe Zhang, Chao Qu, Ying Xin, Xin Jiang

**Affiliations:** 1 Department of Radiation Oncology, The First Hospital of Jilin University, Changchun 130021, China; 2 Department of Internal Medicine, Florida Hospital, Orlando, FL 32803, USA; 3 Key Laboratory of Pathobiology, Ministry of Education, Jilin University, Changchun 130021, China

**Keywords:** oropharyngeal squamous cell carcinoma, human papillomavirus, radiosensitivity, repair, reoxygenation, redistribution, regeneration

## Abstract

Oropharyngeal squamous cell carcinoma (OPSCC) is an important type of head and neck squamous cell carcinoma (HNSCC). The traditional risk factors for OPSCC include carcinogen intake, smoking, alcohol consumption, and lifestyle. In recent years, cases of human papillomavirus (HPV)-related OPSCC have gradually increased. At present, HPV-related OPSCC in developed Western countries comprise up to 90% of all OPSCC cases, while in other developing countries, the proportion of HPV-related OPSCC cases is also gradually increasing. Compared with HPV-negative OPSCC, HPV-positive OPSCC patients have better overall survival rates and local control rates and this improved prognosis may be related to the increased radiosensitivity of HPV-positive tumors. Due to this more favorable prognosis, many downgraded treatment schemes are gradually emerging, including simple radiotherapy instead of concurrent radiotherapy or reduced radiotherapy dose. However, there is insufficient theoretical basis for such schemes. Some studies have shown that delayed repair of DNA damage after radiation, G2/M arrest, increased hypoxia, and decreased proliferation capacity are the main reasons for the increased radiosensitivity of HPV-positive tumor cells. In this review, we discuss the four principles of tumor cell damage caused by radiation, including repair, reoxygenation, redistribution, and regeneration in order to reveal the mechanism whereby HPV increases the radiosensitivity of tumor cells. An attempt was made to provide sufficient information to facilitate more individualized treatment for HPV-positive OPSCC patients, under the premise of good tumor control.

## Introduction

Head and neck cancers (HNCs) are the sixth most common form of cancer worldwide, with approximately 400,000 new cases each year 1. They account for approximately 3.5% of all malignancies in Western countries 2. Head and neck squamous cell carcinoma (HNSCC) accounts for 90% of all HNC cases. Oropharyngeal squamous cell carcinoma (OPSCC) is one of the important types of HNSCC. Patients with OPSCC usually have a poor prognosis, with a 5-year overall survival (OS) rate of less than 50%. There are many causes of OPSCC, but smoking and drinking are generally considered the biggest risk factors. In addition, smoking is closely related to the prognosis of patients with OPSCC 3. However, since Syrjanen et al. suggested that human papillomavirus (HPV) is associated with HNSCC 30 years ago 4, many studies have investigated the relationship between HNSCC and HPV.

HPV infection, like smoking and drinking, has become a risk factor for HNSCC 5, 6. Meanwhile, it has been reported that HPV-related OPSCC patients have higher OS rates and local control rates than HPV-negative patients 7-11. It has been reported that this more favorable prognosis may be attributed to higher radiosensitivity 12-16. In view of this improved prognosis, an increasing number of institutions are attempting a downgraded form of treatment for HPV-positive OPSCC patients 17-20. However, there is insufficient theoretical basis for these revised treatment protocols 21.

Therefore, the purpose of this review is to summarize recent research on HPV-related radiosensitivity and discuss the potential molecular mechanism whereby HPV enhances radiosensitivity.

## The association between HPV and OPSCC

HNC is the sixth most common cancer in the world. Among the different types of HNC, OPSCC is on the rise in some developed countries. In addition to smoking and drinking as risk factors, increasing evidence suggests that HPV infection status is also associated with HNSCC. Currently, HPV-related tumors account for 5-20% of HNSCC cases and 40-90% of OPSCC cases 22-25. The HPV infection rate in tonsillar squamous cell carcinoma is significantly higher than in other subregions 26, 27. HPV infection rates in HNC also vary in different countries. This difference is related to differences in alcohol and tobacco consumption, lifestyle, sexual lifestyle, and the testing methods employed. In China, the rate of HPV-related OPSCC cases is low, which may be related to the large consumption of alcohol and tobacco. However, HPV-positive rates are expected to increase further in China as restrictions on tobacco are implemented.

HPV is a small, double-stranded, circular DNA virus. Currently, there are more than 130 known types of HPV virus, which are divided into high-risk and low-risk groups. The high-risk group mainly included HPV16, 18, 31, 33, 35, 39, 45, 51, 52, 56, 58, 59, 68, 73, 82, etc. The low-risk group mainly included HPV6, 11, 40, 42, 43, 44, 54, 61, 70, 72, 81, etc. HPV-16 and HPV-18 are the most common types in the high-risk group. High-risk HPV infection is usually associated with squamous cell carcinoma (SCC). The most common types in the low-risk group are HPV-6 and HPV-11, which are usually associated with genital warts and papillomas 28. HPV has long been considered an important cause of cervical cancer. It has also been shown to be associated with cancers of the penis, vulva, and upper respiratory tract. Similarly, HPV has been considered as a distinct causative agent and HPV-16 is the most common type associated with OPSCC 29, 30, accounting for 90% of all HPV-positive OPSCC cases 26, 31, 32. HPV infection can lead to abnormalities in the expression of various cycle-related proteins, including increased expression of p16.Some studies have proposed p16 as a surrogate marker for HPV infection 33.

Currently, according to the 8th edition of the American Joint Committee on Cancer (AJCC) Cancer Staging Manual, OPSCC is classified into two different phenotypes, according to the expression of p16^34^. HPV-related OPSCC patients are considered to be a unique group. They are usually younger 35-37, with specific histopathological characteristics, such as HPV-positive tumors that tend to be poorly differentiated and often have basal-cell-like histological structures 28. In addition, HPV-related OPSCC is often associated with degradation of p53, inactivation of the Rb pathway, and upregulation of p16. This is clearly contrasted with tobacco-related OPSCC, which is characterized by p53 mutations and p16 down-regulation 38. It has been reported out that HPV-positive OPSCC has improved survival rates compared with HPV-negative OPSCC 9, 10. Compared with HPV-negative HNSCC patients, HPV-positive HNSCC patients have a 60-80 percent lower risk of dying from cancer 39, 40. This favorable prognosis may be associated with an improved response to radiation or chemotherapy treatment 9, 10, 41-43. It is generally believed that this favorable prognosis is related to the high radiosensitivity of HPV-positive tumors 10, 12-15, 44. In addition, *in vitro* experiments have shown that the average survival rate of HPV-positive cells after radiation is significantly lower than that of HPV-negative cells 14, 16, 45. Therefore, domestic and foreign research institutions are evaluating downgraded forms of treatment for HPV-positive OPSCC patients 17, 46-48, such as simple radiotherapy instead of concurrent chemoradiotherapy and lower radiotherapy dose. This is based on the notion that concurrent chemoradiotherapy is an overtreatment for HPV-positive OPSCC patients 49 and will thus, increase the risk of adverse effects on normal tissues, such as mucosal-related weight loss, bone marrow suppression, and dysphagia, with no difference in tumor control 50-52. As a result, an increasing number of downgraded treatment protocols have been proposed 49, 53-55. Currently, there have been 2 phase III clinical trials showing the same or a similar response to downgraded radiotherapy in patients with stage III-IV HPV-positive OPSCC, when compared to conventional radiotherapy protocols 55, 56. However, the mechanism whereby HPV enhances tumor radiosensitivity is not clear.

Radiosensitivity refers to the rate of death, injury, or other adverse effects on organism, cells, tissues, or organs after receiving ionizing radiation. The radiosensitivity of cells and tissues depends on their biological characteristics and is influenced by environmental factors. The effect of radiation on cells depends on the 4Rs principle in radiobiology, which refers to repair, reoxygenation, redistribution, and regeneration. Therefore, the molecular mechanism responsible for HPV enhancing the radiosensitivity of OPSCC cells is likely related to these four aspects, which is consistent with previous reports. However, most studies have only reported related phenomena, such as the delay in DNA damage repair 15, 45, 57 and G2/M phase arrest 15, 58, 59. Thus far, a clear mechanism to explain how HPV-positive OPSCC has increased radiosensitivity has not been proposed.

Based on the aforementioned studies, HPV has become a recognized risk factor for OPSCC and the proportion of HPV-related OPSCC is increasing year by year. Some studies have preliminarily proposed that HPV-related tumors have high radiosensitivity, which may explain the favorable prognosis of patients with HPV-positive OPSCC (Table 1). Because of this improved prognosis, downgraded forms of treatment for HPV-positive OPSCC patients have been attempted to improve the quality of life of patients and reduce the risk of adverse reactions, while ensuring adequate tumor control.

## The 4Rs principle

### Repair

Several common types of DNA damage caused by radiation include base-pair damage, base-pair loss, cross-linking, and single- or double-strand breakage (DSB). Among these, DSBs are the most common cause of cell death, as all other types of damage are more likely to be repaired. DSBs are usually repaired by one of two mechanisms, non-homologous end-joining (NHEJ) or homologous recombination (HR)^60^. The specific repair mechanism employed depends on the type of damage. If the DNA damage is too severe to repair, eventually, the cell pathway will lead to cell death through apoptosis, aging, or other mechanisms. Therefore, it has been suggested that impairment of DNA repair ability may be the reason why HPV-positive cells are sensitive to radiation 45, 59, 61. Using immunofluorescence staining with anti-γH2AX and 53BP1 antibodies, to detect early signals of cellular responses to DSBs, it has been shown that, in HPV-positive cells, residual γH2AX/53BP1 lesions after irradiation were more obvious, indicating that DBSs remained longer without repair 45. Moreover, HPV was found to be significantly correlated with the radiosensitivity of cells.

Consistent with these results, Prevc et al. evaluated DNA damage repair at various time points after radiation using γH2AX expression as a marker 15. They found that, after a radiation dose of 5 Gy, the amount of γH2AX fluorescence in HPV-positive cells was higher than in HPV-negative cells and remained at a higher level for 24 h. However, 24 h after radiation, γH2AX fluorescence in HPV-negative cells was almost down to normal levels, suggesting that HPV-negative cells repair almost all DNA DSBs within 24 h of radiation exposure. These results showed that the DNA damage repair ability of HPV-positive cells was impaired after radiation, which increased the radiation sensitivity of the cells by 30%. In addition, the impaired repair ability may be related to somatic cell aberrations of DNA repair genes 62. Similarly, in mouse models, the expression of HPV16-E7, leads to a significant increase in γH2AX levels after radiation 61. The latest evidence suggests that the inactivation of Rb delays the resolution of γH2AX lesions, indicating that Rb inactivation may be the cause of impaired DNA repair, particularly by inhibiting NHEJ 63. E7, but not E6, also induces DNA damage, as indicated by the induction of γ-H2AX and 53BP1 nuclear foci, by down-regulating Rb 64. Dok et al. 57 reported a new function of p16 as an HR DNA damage response inhibitor in HPV-positive OPSCC. p16 can damage the DNA repair system by preventing RAD51 from entering the site of DNA damage, thus impeding HR-mediated DNA repair 57.This leads to a shift from homologous to non-homologous repair mechanisms, which induce more errors and cell death. Meanwhile, other studies have observed that p16 can inhibit the formation of RAD51 lesions and increase the frequency of micronuclei. In addition, Jirawatnotai et al. 65 proposed that CCND1, a cell cycle regulatory protein, could directly bind to RAD51 and affect HR. In HPV-positive cells, p16 overexpression can inhibit CCND1. When CCND1 is depleted, RAD51 recruitment to sites of DNA damage decreases, thereby hindering HR and increasing radiosensitivity.

**Figure 1:**
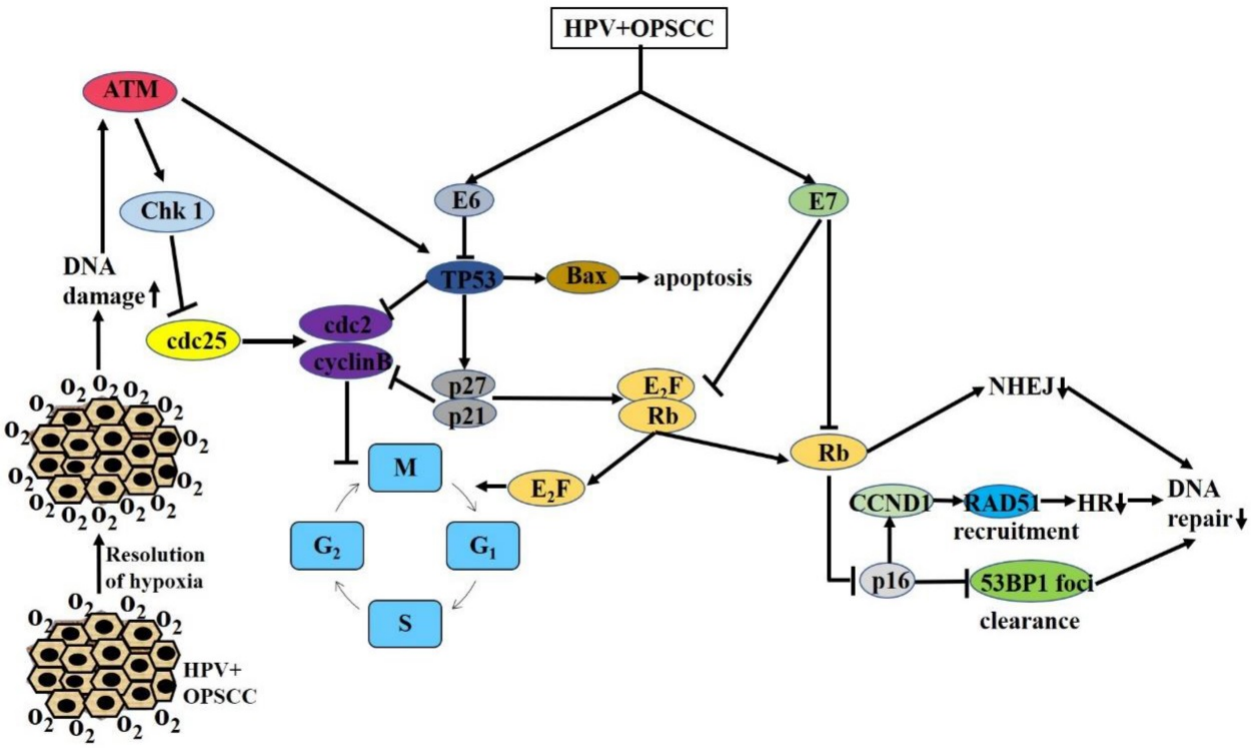
Major mechanisms by which HPV increases OPSCC radiosensitivity: G2/M arrest, resolution of hypoxia, reduced DNA repair

In summary, comparisons of the levels of the DSB signal factors, γH2AX/53BP1, and RAD51 in HPV-positive and HPV-negative cells after radiation have enabled the characterization of DNA damage repair in different cells, further confirming that HPV can increase the radiosensitivity of cells by inhibiting DSB repair (Figure 1).

### Reoxygenation

Radiation causes cells to produce free radicals, which cause damage to DNA. Therefore, oxygen plays an important role in radiation damage and the formation of free radicals. In most solid tumors, hypoxic centers make cancer cells resistant to radiation, while in the DNA microenvironment, free radicals fail to provide oxygen and cause damage to the DNA. Oxygen plays an important role in repairing radiation-induced damage. Thus, under low-oxygen conditions, DNA damage is reduced, leading to cell survival. It has been reported that the radiosensitivity of cells is reduced by 3-fold in a low-oxygen environment. Therefore, studies have indicated that the increased radiosensitivity of HPV-positive cells may be attributed to the improvement of the intracellular hypoxic environment 14, 66. In the Danish Head and Neck Cancer Group (DAHANCA) 5 experiment, HPV-negative cells produced more hypoxia-related modifications than HPV-positive cells, which were closely related to the levels of hypoxia markers 67, 68. In 2018, Lassen et al. concluded that low levels of hypoxia in HPV-positive cells were the reason they avoided the development of radiation resistance 66. In addition, they indicated that the benefit of the hypoxia modifier, nimorazole, was limited to p16-negative tumors, with no effect on p16-positive tumors, suggesting that nimorazole is not effective in HPV-positive cells due to hypoxic deficiency. We also noted that hypoxic regression early in the treatment of HPV-positive tumors may explain the lack of benefit of nimorazole in patients with early regression 69. However, the relationship between hypoxia and HPV is not entirely clear, since several other studies have reported no correlation between hypoxia markers and HPV 70, 71. Meanwhile, Sorensen et al. also showed that oxygen enhancement rate is not different between HPV-positive and HPV-negative cells 72.

In summary, no consistent conclusion has been reached on the relationship between hypoxia and HPV and therefore, the role of hypoxia in HPV-positive cells needs to be further clarified (Figure 1).

### Redistribution

The cell cycle represents the entire life process of a cell, starting from the end of one cell division, through the accumulation of substances, until the end of the next cell division. Most eukaryotic cell cycles consist of four phases, each with different radio-sensitivities. The G2/M phase is the most sensitive phase, followed by G1, and S is the least sensitive phase 73. The sequential cell cycle is the result of the regulation of a series of related genes, and to ensure the normal operation of the cell cycle, there are a series of checkpoints within the cell. When DNA damage occurs, the cell cycle is immediately interrupted and damaged DNA is detected and repaired. If the DNA damage is minor, the damage can be repaired and the cell cycle can return to normal, but if the damage is too severe to repair, it can further initiate apoptosis or other cell death pathways. There are four checkpoints in the cell, including G1/S, S, G2/M, and the mid-to-late checkpoint. The G2/M checkpoint is the most important checkpoint, as it determines cell division and is mainly responsible for detecting DNA damage. Since G2/M phase cells have the highest radiosensitivity, the high radiosensitivity associated with HPV may be due to the G2/M arrest of infected cells, leaving most cells in the G2/M phase, thus causing a large number of cells to be killed 14, 45, 57, 59. In a study of radiation-induced changes in five HPV-positive tumor cell lines, eight cells that were arrested in the G2 phase showed significant changes. Therefore, the authors suggested that the increased radiosensitivity of HPV-positive cells may be related to increased G2/M arrest associated with DSBs 45. Arenz et al. 59 compared the ratio of cells at different cell cycle phases in HPV-positive and HPV-negative cell lines after radiation. They found that HPV-positive cell lines progressed faster at S phase and significantly higher numbers of cells were at G2/M phase. Similarly, Kimple et al. 14 described extensive G2/M arrest in HPV-positive cells. Meanwhile, in 2018, Prevc et al. performed a cell cycle analysis after 15 Gy of radiation and found that HPV-positive cells showed G2 arrest 24 h after radiation and the proportion of G2 cells was significantly higher than that in the control group 15. This phenomenon is also seen in HPV-negative cells, but to a lesser extent. After 72 h of radiation, the proportion of G2 cells in the HPV-positive group decreased to a normal level, possibly as a result of the death of HPV-positive G2 cells during mitosis. In addition, G2 arrest was found to be associated with increased apoptosis and accelerated S phase progression 59.

In summary, several studies have confirmed that HPV can increase the number of cells in G2/M phase and improve the radiosensitivity of tumors by mediating the G2/M arrest of cells after radiation. However, the molecular mechanism whereby HPV participates in G2/M arrest has not been elucidated.

In addition to G2/M arrest after radiation, apoptosis commonly occurs after radiation. Apoptosis, also known as type I programmed cell death, is an active gene-dominated cell death process that plays a key role in normal physiological processes, such as embryogenesis, maintaining the stability of the body's internal environment, and regulating cell proliferation. Apoptosis also refers to cell suicide induced by foreign factors. When DNA damage is severe, cell processes will directly turn to cell death. Radiation may also induce DNA damage in cells to mediate their selective apoptosis through the following three pathways: p53-caspase, p53-Bax-mitochondria-caspase, and the p53-Fas-caspase cascade reaction. p53 can induce apoptosis via exogenous and endogenous pathways. In endogenous pathways, p53 plays a role by activating downstream pro-apoptotic genes, such as *Bax* and *PUMA*, after radiation 74. Bax then inserts into the mitochondrial membrane, causing the release of cytochrome c and triggering apoptosis through the caspase cascade. In exogenous pathways, p53 induces the expression of the apoptotic receptor, CD95/Fas and CS95/Fas ligands, leading to induction of the downstream caspase cascade and ultimately, apoptosis 74. It has been confirmed that the increased radiosensitivity of HPV-positive cells is due to increased levels of p53-mediated apoptosis and cells with high expression levels of wild-type p53 usually have increased radiosensitivity.

It has previously been shown that p53 is usually mutated in HPV-negative tumor cells, whereas wild-type p53 is usually found in HPV-positive tumor cells. In HPV-related OPSCC, although wild-type p53 is partially inactivated by E6 protein, low levels of p53 can still increase the sensitivity of cells to DNA damage factors 7, 74. In addition, studies have shown that HPV-E6-positive OPSCC cell lines have higher radiosensitivity in a p53-independent manner 58. Kimple et al. 14 compared four HPV-positive and HPV-negative cell lines and detected apoptosis by caspase activity measurement and flow cytometry. They found that apoptosis and caspase activity were significantly increased in the HPV-positive group, compared with the HPV-negative group, 24 h after 4 Gy of radiation. Through the detection of cytokine expression, the activation of p53 and its gene expression level were found to be significantly higher in the HPV-positive group after radiation. These results showed that HPV-positive cells promoted apoptosis by activating the p53 pathway, thus increasing the radiosensitivity of the cells. Later, in HPV-positive cell lines, an siRNA was used to knock out the remaining p53, resulting in increased cell colony formation, indicating increased cell survival. Furthermore it was shown that low-level p53 can be activated by radiation, initiating the p53 apoptosis pathway and promoting apoptosis 14.

Previous studies have shown that HPV-E6/E7 can down-regulate p53 and Rb, respectively, resulting in p16 overexpression 38, 75-77. The most common form of DNA damage caused by radiation is DSBs. It has been proposed that the delay in DNA damage repair in HPV-positive cells after radiation leads to the increase in DSBs 15. Molecular biological studies have shown that DSBs can further act on cycle-dependent kinases, including Chk1, Chk2, and cdc25, by activating the ATM/ATR pathway and finally, act on the cdc2-cyclin B complex, leading to G2/M phase arrest. Because G2/M phase cells are most sensitive, cells blocked at G2 phase are killed in large numbers. At the same time, damaged DNA can also stimulate Akt-related pathways and activate the expression of downstream p53, thereby affecting downstream signaling molecules, such as Bcl-2 and Bax, ultimately leading to increased apoptosis. Previous studies have shown that there is a delay in DNA damage repair after radiation and significant G2/M arrest, as well as an increase in the number of apoptotic HPV-positive cells. These studies provide a preliminary explanation for the increased radiosensitivity of HPV-related tumors, including G2/M arrest related to the ATM/ATR pathway and apoptosis related to the Akt-p53 pathway (Figure 1). However, the molecular mechanisms and signaling pathways that cause DNA damage repair delays, cell cycle changes, and increased apoptosis have not been fully elucidated. Therefore, much follow-up basic research is necessary to further clarify the molecular mechanisms.

### Regeneration

Accelerated proliferation refers to the phenomenon of increased proliferation of cells at a certain stage during the radiation process, with different proliferation rates. There are two types of cells that proliferate in the irradiated region. Firstly, some cells travel to the irradiated area for replication from outside the area of irradiation. For example, after radiation injury of oral mucosa and gastrointestinal mucosa, the tissue is repaired by this mechanism. Secondly, cells replicate in the range of exposure. In this way, tumor cells can produce more tumor cells. The accelerated proliferation of tumor cells is not conducive to tumor control and therefore, additional doses of radiation are needed to stop the accelerated proliferation of cells. Therefore, the ability of cells to proliferate after radiation is also one of the factors affecting radiosensitivity.

In an *in vitro* study, the percentage of cells undergoing mitosis significantly decreased in HPV-positive cells, but the proportion of mitotic cells first increased and later declined in HPV-negative cells after radiation. These results suggested that HPV-positive cells have a lower mitotic ability than HPV-negative cells after radiation 15. The EGFR pathway is the most commonly upregulated mitotic signaling pathway in HNSCC and is usually associated with therapeutic resistance and poor prognosis. Previous studies have shown that the expression levels of HPV and EGFR are negatively correlated in OPSCC 78. Gupta et al. 79 showed that EGFR phosphorylation is increased in HPV-negative cell lines with strong radiation resistance. In addition, this activated EGFR then activates the oncogene, mTOR, through the Akt pathway.

Cancer stem cells (CSCs) are the reserve cells in tumor tissues. When cells are damaged, CSCs will immediately enter the differentiation process and supplement the damaged tumor cells. Therefore, previous studies have suggested that CSCs may be one of the causes of tumor radiation resistance. Some studies have reported that the lack of CSCs in HPV-positive tumors may be the reason for its increased radiosensitivity 80. Michelle et al. 80 studied a total of 711 patients with OPSCC. The presence and intensity of CD44 and CD98 (two CSC markers), determined by immunohistochemistry, was used to assess the proportion of CSCs. Their results showed that the expression levels of CD44 and CD98 were lower in HPV-positive cells than in HPV-negative cells. The amount of CD44 staining and the proportion of CD44-positive cells in HPV-negative tumors were significantly higher than in HPV-positive tumors. CD98 staining intensity and the CD98-positive cell ratio of HPV-negative tumors were also significantly higher than those of HPV-positive tumors. This study confirmed that fewer cells expressed the CSC-enrichment markers, CD44 and CD98, in HPV-positive OPSCC, which may be the reason for the higher radiosensitivity 80.

Although a positive effect of HPV on the prognosis of OPSCC patients has been demonstrated, methods for diagnosing HPV infection have not been developed. Commonly used diagnostic methods include the detection of HPV-DNA by polymerase chain reaction; the detection of the alternative marker, p16, by immunohistochemistry (IHC); and *in situ* hybridization (ISH). In addition, the Linear Array HPV Genotyping Test is a newly developed Test. The sensitivity and specificity of IHC are 94-97% and 83%-84%, respectively, while the sensitivity and specificity of ISH are 85-88% and 88-95%, respectively 81-83. Different testing methods can lead to different results and the uncertainty of HPV status can affect the prognosis of OPSCC patients. Currently, there is no test with high sensitivity and specificity and therefore, the latest National Comprehensive Cancer Network (NCCN) guidelines recommend comprehensive diagnosis of HPV infection by more than one method.

## Conclusion

In summary, the more favorable prognosis of HPV-positive OPSCC patients may be attributed to high radiosensitivity, but the specific molecular mechanism has not been fully elucidated. Therefore, it is necessary to perform further basic research on this topic. Elucidating the molecular mechanism and related pathways of HPV enhancing radiosensitivity in tumor cells will provide a new understanding of HPV-related OPSCC. In addition to tumor control, treatment-related adverse reactions have also attracted more and more attention. Confirmation of high radiosensitivity of HPV related OPSCC will provide theoretical basis for the implementation of downgrading treatment. This will provide more individualized treatment for HPV positive patient and provide new targets for the treatment of OPSCC.

## Figures and Tables

**Table 1:** Summary of the mechanisms of increased radiosensitivity

No.	Author	Detection method	Detection indicators	Conclusion	Year	Journal
1	Prevc, A.^15^	γ-H2AX Assay	γ-H2AX	DNA damage repair capacity is impaired	2018	Radiat Res
2	Rieckmann, T.^45^	Immunofluorescence staining	γ-H2AX, 53BP1		2013	Radiother Oncol
3	Dok, R.^57^	immunostaining	RAD51, cyclin D1		2014	Cancer Res
4	Arenz, A.^59^	Western blot	DSB level		2014	Strahlenther Onkol
5	Park, J. W.^61^	immunoblotting	γ-H2AX, RAD51		2014	Radiother Oncol
6	Carr, S. M.^63^	Western blot	53BP1, pRb		2014	Proc Natl Acad Sci
7	Lassen, P.^66^	Tumor control between nimorazole treatment and control	Local control rate	Increased radiosensitivity due to hypoxic conditions improvement	2010	Radiother Oncol
8	Overgaard, J.^67^	ELISA	osteopontin (SPP1),		2005	Lancet Oncol
9	Toustrup, K.	Quantify Gene expressions	LRC , DSS		2012	Radiother Oncol
10	Kimple, R. J.^14^	Flow cytometry	cell cycle distribution	The increased sensitivity of HPV+OPSCC is due to G2/M arrest	2013	Cancer Res
11	Prevc, A.^15^	Flow cytometry	Number of cells in each period		2018	Radiat Res
12	Rieckmann, T.^45^	the G1-efflux assay,	G1 and G2 arrest,		2013	Radiother Oncol
13	Arenz, A.^59^	Flow cytometry	Number of cells in each period		2014	Strahlenther Onkol
14	Kimple, R. J.^14^	A luminescent DEVD cleavage assay, Flow cytometry	Caspase 3/7 activity	Increased apoptosis of HPV+ cells	2013	Cancer Res
15	Prevc, A.^15^	Microscopic counting	the number of mitotic cells	HPV+ cells have weak mitosis after radiation	2018	Radiat Res
16	Mirghani, H.^78^	--	EGFR		2014	Eur J Cancer
17	Rietbergen, M.M.^80^	Immunostaining, PCR	CD44,CD98		2014	Eur J Cancer

## Abbreviations

AJCC: American Joint Committee on Cancer
CSCs: cancer stem cells
DSB: double-strand breakage
HNCs: head and neck cancers
HNSCC: head and neck squamous cell carcinoma
HR: homologous recombination
HPV: human papillomavirus
IHC: immunohistochemistry
ISH: in situ hybridization
NCCN: National Comprehensive Cancer Network
NHEJ: non-homologous end-joining
OPSCC: oropharyngeal squamous cell carcinoma
OS: overall survival
SCC: Squamous cell carcinoma

## References

[1] Duray A, Descamps G, Decaestecker C, Remmelink M, Sirtaine N, Lechien J (2012). Human papillomavirus DNA strongly correlates with a poorer prognosis in oral cavity carcinoma. The Laryngoscope.

[2] Ferlay J, Parkin DM, Steliarova-Foucher E (2010). Estimates of cancer incidence and mortality in Europe in 2008. European journal of cancer (Oxford, England: 1990).

[3] Browman GP, Mohide EA, Willan A, Hodson I, Wong G, Grimard L (2002). Association between smoking during radiotherapy and prognosis in head and neck cancer: a follow-up study. Head & neck.

[4] Syrjanen K, Syrjanen S, Pyrhonen S (1982). Human papilloma virus (HPV) antigens in lesions of laryngeal squamous cell carcinomas. ORL; journal for oto-rhino-laryngology and its related specialties.

[5] Gillison ML, Koch WM, Capone RB, Spafford M, Westra WH, Wu L (2000). Evidence for a causal association between human papillomavirus and a subset of head and neck cancers. Journal of the National Cancer Institute.

[6] Klussmann JP, Weissenborn S, Fuchs PG (2001). Human papillomavirus infection as a risk factor for squamous-cell carcinoma of the head and neck. The New England journal of medicine.

[7] Licitra L, Perrone F, Bossi P, Suardi S, Mariani L, Artusi R (2006). High-risk human papillomavirus affects prognosis in patients with surgically treated oropharyngeal squamous cell carcinoma. Journal of clinical oncology: official journal of the American Society of Clinical Oncology.

[8] O'Rorke MA, Ellison MV, Murray LJ, Moran M, James J, Anderson LA (2012). Human papillomavirus related head and neck cancer survival: a systematic review and meta-analysis. Oral oncology.

[9] Rischin D, Young RJ, Fisher R, Fox SB, Le QT, Peters LJ (2010). Prognostic significance of p16INK4A and human papillomavirus in patients with oropharyngeal cancer treated on TROG 02.02 phase III trial. Journal of clinical oncology: official journal of the American Society of Clinical Oncology.

[10] Fakhry C, Westra WH, Li S, Cmelak A, Ridge JA, Pinto H (2008). Improved survival of patients with human papillomavirus-positive head and neck squamous cell carcinoma in a prospective clinical trial. Journal of the National Cancer Institute.

[11] Lu DJ, Luu M, Mita A, Scher K, Shiao SL, Yoshida EP (2018). Human papillomavirus-associated oropharyngeal cancer among patients aged 70 and older: Dramatically increased prevalence and clinical implications. European journal of cancer (Oxford, England: 1990).

[12] Chen AM, Zahra T, Daly ME, Farwell DG, Luu Q, Gandour-Edwards R (2013). Definitive radiation therapy without chemotherapy for human papillomavirus-positive head and neck cancer. Head & neck.

[13] Bossi P, Orlandi E, Miceli R, Perrone F, Guzzo M, Mariani L (2014). Treatment-related outcome of oropharyngeal cancer patients differentiated by HPV dictated risk profile: a tertiary cancer centre series analysis. Annals of oncology: official journal of the European Society for Medical Oncology.

[14] Kimple RJ, Smith MA, Blitzer GC, Torres AD, Martin JA, Yang RZ (2013). Enhanced radiation sensitivity in HPV-positive head and neck cancer. Cancer research.

[15] Prevc A, Kranjc S, Cemazar M, Todorovic V, Zegura B, Novak M (2018). Dose-Modifying Factor of Radiation Therapy with Concurrent Cisplatin Treatment in HPV-Positive Squamous Cell Carcinoma: A Preclinical Study. Radiation research.

[16] Zhang M, Rose B, Lee CS, Hong AM (2015). In vitro 3-dimensional tumor model for radiosensitivity of HPV positive OSCC cell lines. Cancer biology & therapy.

[17] Tsai CJ, McBride SM, Riaz N, Lee NY (2019). Reducing the Radiation Therapy Dose Prescription for Elective Treatment Areas in Human Papillomavirus-Associated Oropharyngeal Carcinoma Being Treated With Primary Chemoradiotherapy at Memorial Sloan Kettering Cancer Center. Practical radiation oncology.

[18] Psyrri A, Rampias T, Vermorken JB (2014). The current and future impact of human papillomavirus on treatment of squamous cell carcinoma of the head and neck. Annals of oncology: official journal of the European Society for Medical Oncology.

[19] Kofler B, Laban S, Busch CJ, Lorincz B, Knecht R (2014). New treatment strategies for HPV-positive head and neck cancer. European archives of oto-rhino-laryngology: official journal of the European Federation of Oto-Rhino-Laryngological Societies (EUFOS): affiliated with the German Society for Oto-Rhino-Laryngology - Head and Neck Surgery.

[20] Mehanna H (2017). Update on De-intensification and Intensification Studies in HPV. Recent results in cancer research Fortschritte der Krebsforschung Progres dans les recherches sur le cancer.

[21] Howard J, Dwivedi RC, Masterson L, Kothari P, Quon H, Holsinger FC (2018). De-intensified adjuvant (chemo)radiotherapy versus standard adjuvant chemoradiotherapy post transoral minimally invasive surgery for resectable HPV-positive oropharyngeal carcinoma. The Cochrane database of systematic reviews.

[22] Zaravinos A (2014). An updated overview of HPV-associated head and neck carcinomas. Oncotarget.

[23] Chaturvedi AK, Engels EA, Pfeiffer RM, Hernandez BY, Xiao W, Kim E (2011). Human papillomavirus and rising oropharyngeal cancer incidence in the United States. Journal of clinical oncology: official journal of the American Society of Clinical Oncology.

[24] Gillison ML, Broutian T, Pickard RK, Tong ZY, Xiao W, Kahle L (2012). Prevalence of oral HPV infection in the United States, 2009-2010. Jama.

[25] Nasman A, Attner P, Hammarstedt L, Du J, Eriksson M, Giraud G (2009). Incidence of human papillomavirus (HPV) positive tonsillar carcinoma in Stockholm, Sweden: an epidemic of viral-induced carcinoma?. International journal of cancer.

[26] Venuti A, Badaracco G, Rizzo C, Mafera B, Rahimi S, Vigili M (2004). Presence of HPV in head and neck tumours: high prevalence in tonsillar localization. Journal of experimental & clinical cancer research: CR.

[27] Koskinen WJ, Chen RW, Leivo I, Makitie A, Back L, Kontio R (2003). Prevalence and physical status of human papillomavirus in squamous cell carcinomas of the head and neck. International journal of cancer.

[28] Munoz N, Bosch FX, de Sanjose S, Herrero R, Castellsague X, Shah KV (2003). Epidemiologic classification of human papillomavirus types associated with cervical cancer. The New England journal of medicine.

[29] Kreimer AR, Clifford GM, Boyle P, Franceschi S (2005). Human papillomavirus types in head and neck squamous cell carcinomas worldwide: a systematic review. Cancer epidemiology, biomarkers & prevention: a publication of the American Association for Cancer Research, cosponsored by the American Society of Preventive Oncology.

[30] Herrero R, Castellsague X, Pawlita M, Lissowska J, Kee F, Balaram P (2003). Human papillomavirus and oral cancer: the International Agency for Research on Cancer multicenter study. Journal of the National Cancer Institute.

[31] Bouvard V, Baan R, Straif K, Grosse Y, Secretan B, El Ghissassi F (2009). A review of human carcinogens-Part B: biological agents. The Lancet Oncology.

[32] Stransky N, Egloff AM, Tward AD, Kostic AD, Cibulskis K, Sivachenko A (2011). The mutational landscape of head and neck squamous cell carcinoma. Science (New York, NY).

[33] Lewis JS Jr (2012). p16 Immunohistochemistry as a standalone test for risk stratification in oropharyngeal squamous cell carcinoma. Head and neck pathology.

[34] van Gysen K, Stevens M, Guo L, Jayamanne D, Veivers D, Wignall A (2019). Validation of the 8(th) edition UICC/AJCC TNM staging system for HPV associated oropharyngeal cancer patients managed with contemporary chemo-radiotherapy. BMC cancer.

[35] Lajer CB, von Buchwald C (2010). The role of human papillomavirus in head and neck cancer. APMIS: acta pathologica, microbiologica, et immunologica Scandinavica.

[36] Snow AN, Laudadio J (2010). Human papillomavirus detection in head and neck squamous cell carcinomas. Advances in anatomic pathology.

[37] D'Souza G, Zhang HH, D'Souza WD, Meyer RR, Gillison ML (2010). Moderate predictive value of demographic and behavioral characteristics for a diagnosis of HPV16-positive and HPV16-negative head and neck cancer. Oral oncology.

[38] Marur S, D'Souza G, Westra WH, Forastiere AA (2010). HPV-associated head and neck cancer: a virus-related cancer epidemic. The Lancet Oncology.

[39] Weinberger PM, Yu Z, Haffty BG, Kowalski D, Harigopal M, Brandsma J (2006). Molecular classification identifies a subset of human papillomavirus-associated oropharyngeal cancers with favorable prognosis. Journal of clinical oncology: official journal of the American Society of Clinical Oncology.

[40] Ragin CC, Taioli E (2007). Survival of squamous cell carcinoma of the head and neck in relation to human papillomavirus infection: review and meta-analysis. International journal of cancer.

[41] Petrelli F, Sarti E, Barni S (2014). Predictive value of human papillomavirus in oropharyngeal carcinoma treated with radiotherapy: An updated systematic review and meta-analysis of 30 trials. Head & neck.

[42] Ang KK, Harris J, Wheeler R, Weber R, Rosenthal DI, Nguyen-Tan PF (2010). Human papillomavirus and survival of patients with oropharyngeal cancer. The New England journal of medicine.

[43] Fakhry C, Zhang Q, Nguyen-Tan PF, Rosenthal D, El-Naggar A, Garden AS (2014). Human papillomavirus and overall survival after progression of oropharyngeal squamous cell carcinoma. Journal of clinical oncology: official journal of the American Society of Clinical Oncology.

[44] Lindel K, Beer KT, Laissue J, Greiner RH, Aebersold DM (2001). Human papillomavirus positive squamous cell carcinoma of the oropharynx: a radiosensitive subgroup of head and neck carcinoma. Cancer.

[45] Rieckmann T, Tribius S, Grob TJ, Meyer F, Busch CJ, Petersen C (2013). HNSCC cell lines positive for HPV and p16 possess higher cellular radiosensitivity due to an impaired DSB repair capacity. Radiotherapy and oncology: journal of the European Society for Therapeutic Radiology and Oncology.

[46] Lassen P, Eriksen JG, Krogdahl A, Therkildsen MH, Ulhoi BP, Overgaard M (2011). The influence of HPV-associated p16-expression on accelerated fractionated radiotherapy in head and neck cancer: evaluation of the randomised DAHANCA 6&7 trial. Radiotherapy and oncology: journal of the European Society for Therapeutic Radiology and Oncology.

[47] Stock GT, Bonadio R, de Castro GJ (2018). De-escalation treatment of human papillomavirus-positive oropharyngeal squamous cell carcinoma: an evidence-based review for the locally advanced disease. Current opinion in oncology.

[48] Suton P, Skelin M, Rakusic Z, Dokuzovic S, Luksic I (2019). Cisplatin-based chemoradiotherapy vs. cetuximab-based bioradiotherapy for p16-positive oropharyngeal cancer: an updated meta-analysis including trials RTOG 1016 and De-ESCALaTE. European archives of oto-rhino-laryngology: official journal of the European Federation of Oto-Rhino-Laryngological Societies (EUFOS): affiliated with the German Society for Oto-Rhino-Laryngology - Head and Neck Surgery.

[49] O'Sullivan B, Huang SH, Siu LL, Waldron J, Zhao H, Perez-Ordonez B (2013). Deintensification candidate subgroups in human papillomavirus-related oropharyngeal cancer according to minimal risk of distant metastasis. Journal of clinical oncology: official journal of the American Society of Clinical Oncology.

[50] Forastiere AA (2008). Chemotherapy in the treatment of locally advanced head and neck cancer. Journal of surgical oncology.

[51] Leclerc M, Maingon P, Hamoir M, Dalban C, Calais G, Nuyts S (2013). A dose escalation study with intensity modulated radiation therapy (IMRT) in T2N0, T2N1, T3N0 squamous cell carcinomas (SCC) of the oropharynx, larynx and hypopharynx using a simultaneous integrated boost (SIB) approach. Radiotherapy and oncology: journal of the European Society for Therapeutic Radiology and Oncology.

[52] Denis F, Garaud P, Bardet E, Alfonsi M, Sire C, Germain T (2003). Late toxicity results of the GORTEC 94-01 randomized trial comparing radiotherapy with concomitant radiochemotherapy for advanced-stage oropharynx carcinoma: comparison of LENT/SOMA, RTOG/EORTC, and NCI-CTC scoring systems. International journal of radiation oncology, biology, physics.

[53] Masterson L, Moualed D, Liu ZW, Howard JE, Dwivedi RC, Tysome JR (2014). De-escalation treatment protocols for human papillomavirus-associated oropharyngeal squamous cell carcinoma: a systematic review and meta-analysis of current clinical trials. European journal of cancer (Oxford, England: 1990).

[54] Kimple RJ, Harari PM (2014). Is radiation dose reduction the right answer for HPV-positive head and neck cancer?. Oral oncology.

[55] Chen AM, Felix C, Wang PC, Hsu S, Basehart V, Garst J (2017). Reduced-dose radiotherapy for human papillomavirus-associated squamous-cell carcinoma of the oropharynx: a single-arm, phase 2 study. The Lancet Oncology.

[56] Marur S, Li S, Cmelak AJ, Gillison ML, Zhao WJ, Ferris RL (2017). E1308: Phase II Trial of Induction Chemotherapy Followed by Reduced-Dose Radiation and Weekly Cetuximab in Patients With HPV-Associated Resectable Squamous Cell Carcinoma of the Oropharynx- ECOG-ACRIN Cancer Research Group. Journal of clinical oncology: official journal of the American Society of Clinical Oncology.

[57] Dok R, Kalev P, Van Limbergen EJ, Asbagh LA, Vazquez I, Hauben E (2014). p16INK4a impairs homologous recombination-mediated DNA repair in human papillomavirus-positive head and neck tumors. Cancer research.

[58] Pang E, Delic NC, Hong A, Zhang M, Rose BR, Lyons JG (2011). Radiosensitization of oropharyngeal squamous cell carcinoma cells by human papillomavirus 16 oncoprotein E6 *I. International journal of radiation oncology, biology, physics.

[59] Arenz A, Ziemann F, Mayer C, Wittig A, Dreffke K, Preising S (2014). Increased radiosensitivity of HPV-positive head and neck cancer cell lines due to cell cycle dysregulation and induction of apoptosis. Strahlentherapie und Onkologie: Organ der Deutschen Rontgengesellschaft [et al].

[60] Kim BM, Hong Y, Lee S, Liu P, Lim JH, Lee YH (2015). Therapeutic Implications for Overcoming Radiation Resistance in Cancer Therapy. International journal of molecular sciences.

[61] Park JW, Nickel KP, Torres AD, Lee D, Lambert PF, Kimple RJ (2014). Human papillomavirus type 16 E7 oncoprotein causes a delay in repair of DNA damage. Radiotherapy and oncology: journal of the European Society for Therapeutic Radiology and Oncology.

[62] Seiwert TY, Zuo Z, Keck MK, Khattri A, Pedamallu CS, Stricker T (2015). Integrative and comparative genomic analysis of HPV-positive and HPV-negative head and neck squamous cell carcinomas. Clinical cancer research: an official journal of the American Association for Cancer Research.

[63] Carr SM, Munro S, Zalmas LP, Fedorov O, Johansson C, Krojer T (2014). Lysine methylation-dependent binding of 53BP1 to the pRb tumor suppressor. Proceedings of the National Academy of Sciences of the United States of America.

[64] Park JW, Shin MK, Lambert PF (2014). High incidence of female reproductive tract cancers in FA-deficient HPV16-transgenic mice correlates with E7's induction of DNA damage response, an activity mediated by E7's inactivation of pocket proteins. Oncogene.

[65] Jirawatnotai S, Hu Y, Michowski W, Elias JE, Becks L, Bienvenu F (2011). A function for cyclin D1 in DNA repair uncovered by protein interactome analyses in human cancers. Nature.

[66] Lassen P, Eriksen JG, Hamilton-Dutoit S, Tramm T, Alsner J, Overgaard J (2010). HPV-associated p16-expression and response to hypoxic modification of radiotherapy in head and neck cancer. Radiotherapy and oncology: journal of the European Society for Therapeutic Radiology and Oncology.

[67] Overgaard J, Eriksen JG, Nordsmark M, Alsner J, Horsman MR (2005). Plasma osteopontin, hypoxia, and response to the hypoxia sensitiser nimorazole in radiotherapy of head and neck cancer: results from the DAHANCA 5 randomised double-blind placebo-controlled trial. The Lancet Oncology.

[68] Toustrup K, Sorensen BS, Lassen P, Wiuf C, Alsner J, Overgaard J (2012). Gene expression classifier predicts for hypoxic modification of radiotherapy with nimorazole in squamous cell carcinomas of the head and neck. Radiotherapy and oncology: journal of the European Society for Therapeutic Radiology and Oncology.

[69] Lee N, Schoder H, Beattie B, Lanning R, Riaz N, McBride S (2016). Strategy of Using Intratreatment Hypoxia Imaging to Selectively and Safely Guide Radiation Dose De-escalation Concurrent With Chemotherapy for Locoregionally Advanced Human Papillomavirus-Related Oropharyngeal Carcinoma. International journal of radiation oncology, biology, physics.

[70] Mortensen LS, Johansen J, Kallehauge J, Primdahl H, Busk M, Lassen P (2012). FAZA PET/CT hypoxia imaging in patients with squamous cell carcinoma of the head and neck treated with radiotherapy: results from the DAHANCA 24 trial. Radiotherapy and oncology: journal of the European Society for Therapeutic Radiology and Oncology.

[71] Jansen JF, Carlson DL, Lu Y, Stambuk HE, Moreira AL, Singh B (2012). Correlation of a priori DCE-MRI and (1)H-MRS data with molecular markers in neck nodal metastases: Initial analysis. Oral oncology.

[72] Sorensen BS, Busk M, Olthof N, Speel EJ, Horsman MR, Alsner J (2013). Radiosensitivity and effect of hypoxia in HPV positive head and neck cancer cells. Radiotherapy and oncology: journal of the European Society for Therapeutic Radiology and Oncology.

[73] Dayal R, Singh A, Pandey A, Mishra KP (2014). Reactive oxygen species as mediator of tumor radiosensitivity. Journal of cancer research and therapeutics.

[74] Kostareli E, Holzinger D, Hess J (2012). New Concepts for Translational Head and Neck Oncology: Lessons from HPV-Related Oropharyngeal Squamous Cell Carcinomas. Frontiers in oncology.

[75] Samuels SE, Eisbruch A, Beitler JJ, Corry J, Bradford CR, Saba NF (2016). Management of locally advanced HPV-related oropharyngeal squamous cell carcinoma: where are we?. European archives of oto-rhino-laryngology: official journal of the European Federation of Oto-Rhino-Laryngological Societies (EUFOS): affiliated with the German Society for Oto-Rhino-Laryngology - Head and Neck Surgery.

[76] Andl T, Kahn T, Pfuhl A, Nicola T, Erber R, Conradt C (1998). Etiological involvement of oncogenic human papillomavirus in tonsillar squamous cell carcinomas lacking retinoblastoma cell cycle control. Cancer research.

[77] Li W, Thompson CH, Cossart YE, O'Brien CJ, McNeil EB, Scolyer RA (2004). The expression of key cell cycle markers and presence of human papillomavirus in squamous cell carcinoma of the tonsil. Head & neck.

[78] Mirghani H, Amen F, Moreau F, Guigay J, Hartl DM, Lacau St Guily J (2014). Oropharyngeal cancers: relationship between epidermal growth factor receptor alterations and human papillomavirus status. European journal of cancer (Oxford, England: 1990).

[79] Gupta AK, Lee JH, Wilke WW, Quon H, Smith G, Maity A (2009). Radiation response in two HPV-infected head-and-neck cancer cell lines in comparison to a non-HPV-infected cell line and relationship to signaling through AKT. International journal of radiation oncology, biology, physics.

[80] Rietbergen MM, Martens-de Kemp SR, Bloemena E, Witte BI, Brink A, Baatenburg de Jong RJ (2014). Cancer stem cell enrichment marker CD98: a prognostic factor for survival in patients with human papillomavirus-positive oropharyngeal cancer. European journal of cancer.

[81] Jordan RC, Lingen MW, Perez-Ordonez B, He X, Pickard R, Koluder M (2012). Validation of methods for oropharyngeal cancer HPV status determination in US cooperative group trials. The American journal of surgical pathology.

[82] Cantley RL, Gabrielli E, Montebelli F, Cimbaluk D, Gattuso P, Petruzzelli G (2011). Ancillary studies in determining human papillomavirus status of squamous cell carcinoma of the oropharynx: a review. Pathology research international.

[83] Prigge ES, Arbyn M, von Knebel Doeberitz M, Reuschenbach M (2017). Diagnostic accuracy of p16(INK4a) immunohistochemistry in oropharyngeal squamous cell carcinomas: A systematic review and meta-analysis. International journal of cancer.

